# A complex interaction between pre-harvest and post-harvest factors determines fresh-cut melon quality and aroma

**DOI:** 10.1038/s41598-019-39196-0

**Published:** 2019-02-26

**Authors:** Natasha D. Spadafora, Giacomo Cocetta, Marina Cavaiuolo, Roberta Bulgari, Rakhee Dhorajiwala, Antonio Ferrante, Anna Spinardi, Hilary J. Rogers, Carsten T. Müller

**Affiliations:** 1 School of Biosciences, Cardiff University, Sir Martin Evans Building, Museum Avenue, Cardiff, CF10 3AX United Kingdom; 2 Markes International Ltd, Gwaun Elai Medi-Science Campus, Llantrisant, RCT CF72 8XL United Kingdom; 3 Department of Agricultural and Environmental Sciences, Università degli Studi di Milano, Via Celoria 2, 20133 Milano, Italy; 4 Present Address: Institut de Biologie Physico-Chimique, Paris, France

## Abstract

Melons are prized for their characteristic aroma, however, pre-harvest growth, stage of ripening at harvest, post-harvest processing and storage conditions lead to quality changes in fresh-cut fruit. We considered changes in metabolites and gene expression over 14 days storage to assess underlying mechanisms and identify potential quality markers. Overall, 99 volatile organic compounds (VOCs) were detected and VOC profiles discriminated between two melon seasons, cut-size, storage temperatures and storage time, although season affected their discriminatory power. Abundance of two VOCs fell rapidly and was not associated with cut size, indicating their use as markers for early changes post-processing. Non-acetate to acetate ester ratio differed between the seasons and correlated with changes in alcohol acyl-transferase (*CmAAT1*) gene expression. Furthermore, *CmAAT1* expression clustered with two ester VOCs that may be potential new products of this enzyme. Season also strongly affected post-harvest sugar content, most likely attributable to meteorological differences during growth. Storage temperature and cut size affected expression of transcription factors *ERF71*, *ERF106*, and *TINY*, whose expression generally rose during storage, probably related to increased stress. Thus, although time × temperature of storage are key factors, pre-harvest conditions and fruit processing impact significantly gene expression and aroma loss post-harvest.

## Introduction

Commercialization and consumption of ready to eat cut melons is an increasing market^1^. Melons are valued for their taste and characteristic aroma, which is particularly associated with the climacteric aromatic varieties^2–5^. However, processed melon portions are highly perishable with a shelf life of up to 10–15 days at 2 °C^6^, although at higher temperatures many quality and nutritional parameters decline much more rapidly^1,7^. Preparation for fresh fruit salads involves wounding the fruit flesh, triggering a series of physiological events that can lead to a severe loss of product quality^8^. These physiological processes can be slowed down by lowering the storage temperature^7^. Consequently, cold storage at a recommended temperature of 0–5 °C is essential throughout processing and in the supply chain^9,10^. In addition, pre-harvest factors are also known to have a significant impact on post-harvest quality of fresh cut produce^11^ including soil composition, watering and light levels.

Fruit deterioration leads to increased reactive oxygen species (ROS) and consequent loss of membrane integrity, which can be assessed by measurement of lipid peroxidation^12^. Wounding also elicits the production of ROS^13^ as well as volatile organic compounds (VOCs), secondary metabolites, and ethylene^11^. Moreover, the classes of VOCs that are produced in response to wounding differ depending on fruit ripeness^14^. Temperature of storage also affects flavour (sweetness) and aroma (VOCs), of critical importance to the consumer^15,16^. In response to post-harvest stress, sugar content falls as sugars represent the main substrate for respiratory energy production^17^. Sugars can also be partially fermented, generating off flavours and compromising the sensory quality of melon^18^.

The main volatiles produced by melon fruit are esters, sulphur-containing aromatic compounds, short-chain alcohols and aldehydes, sesquiterpenes and norisoprenes^19^. However, the specific VOCs produced by melon are dependent on stage of maturity^19^. Moreover, in several Cantaloupe cultivars, and across different years and seasons there was an increase in non-acetate/acetate volatile ester ratio during storage^15,16,20^. The change in ester ratio may account for altered flavour, and depend on temperature of storage^7^. Processing also affects the VOC profile in melons: a rapid loss of esters was found in thin slices^10^ but not thicker slices^16^. Changes in VOC profiles and sugars can be detrimental to the sale of the fresh-cut melon^21^.

Some post-harvest changes are the result of altered gene expression. Changes in a large number of *EREBP* transcription factor family genes were noted during fruit ripening^22^. One of these, *CmERF2*, was also up-regulated in response to cold stress in leaves^23^. In contrast, *CmERF1*, which is not up-regulated during fruit ripening, was up-regulated in leaves in response to drought and salt stress treatments. A *DREB* gene with homology to the Arabidopsis *TINY* gene was also strongly up-regulated in response to jasmonate in melon leaves^23^. During post-harvest processing and storage of fruit, the majority of the cells are still metabolically active and therefore responses to the stresses imposed are likely to involve changes in gene expression, although very few studies are available^24^.

A key gene linked to VOC production in melon fruit is alcohol acyl-transferase (*CmAAT1*) that catalyses the formation of esters from a range of alcohols and acyl-CoA substrates^25^ with a preference for the formation of E-2-hexenyl acetate and hexyl hexanoate^26^. *CmAAT1* expression increases with fruit ripening and is linked to ethylene production. Other members of the *CmAAT* gene family differ in their substrate specificity, and until recently, CmAAT2 was thought to be inactive. However, recent metabolomic analyses^27^ suggest that this enzyme may be involved in the biosynthesis of several ethyl, thio and thioethyl esters.

Data are presented here on the complex interaction amongst different factors affecting fresh cut melon quality: cut size, time and temperature of storage, and seasonal effects. Different cut sizes were selected to assess effects of wounding and surface area to volume ratio. Time x temperature of storage was assessed using a shorter time interval at the higher compared to the lower temperature to reflect the greatly accelerated metabolism at 20 °C compared to 4 °C. Analysis of VOCs shows that they can discriminate amongst all these parameters, reflecting changes at the biochemical and gene expression levels and providing useful markers for assessing fresh cut fruit quality.

## Results

### Season, treatment and storage time affected the VOC profile

A total of 99 VOCs were quantifiable across all samples of two experiments testing cut size (1 × 1 cm or 3 × 2 cm) and temperature of storage (20 °C or 4 °C) over all time points (up to 4 days at 20 °C and 14 days at 4 °C) and across two seasons (2013 and 2014). Of these, 87 could be identified putatively, based on their mass spectral and retention index match to the NIST database (Supplementary Table S1). These included 23 acetate esters and 38 non-acetate esters, seven sulphur containing compounds, four alcohols, two terpenes, two alkanes, two acids, two aldehydes and single compounds representing alkenes, amines, anhydrides, oxiranes, aromatic compounds, ketones and nitriles. However, only 27 VOCs were present in both years of both experiments. There was also a difference in the total number of VOCs detected in the two seasons: the greatest number of different VOCs was found in the 2014 (81) and the least in the 2013 (49) two-temperature experiments, while similar numbers of different VOCs were found in the 2013 (65) and 2014 (62) cut size experiments. A comparison of VOC chemical classes between the two seasons (Table 1), revealed significant differences in the proportions in six classes of VOCs between the two seasons in the temperature experiment and seven in the cut size experiment, including acids, alcohols, oxiranes, and terpenes. Notably the relative abundance of acetate and non-acetate esters between the two years in the cut experiment differed significantly, while it was not affected by season in the storage temperature experiment.

**Table 1:** Comparison of the mean % abundance of VOC families and abundance of metabolites across all samples between the two seasons in two temperatures (t) and the cut size (c) experiments (Kruskal Wallis).

VOC classes^a^	2013t	2014t	*P* value	2013c	2014c	*P* value
Acetate esters	50.22 ± 3.81	49.02 ± 8.62	n.s.	36.99 ± 2.47	62.71 ± 14.3	***
Acids	0.313 ± 0.562	1.38 ± 0.850	***	0.150 ± 0.274	1.540 ± 1.43	***
Alcohols	0.110 ± 0.046	0.206 ± 0.166	**	9.25 ± 2.54	0.116 ± 0.078	***
Aldehydes	0.014 ± 0.013	0.037 ± 0.034	**	0.008 ± 0.010	0.018 ± 0.033	ns
Alkanes	0	0.008 ± 0.010	n.a.	0	0.002 ± 0.008	n.a.
Amines	0.018 ± 0.035	0.022 ± 0.046	n.s.	0	0	n.a.
Anhydrides	2.23 ± 3.49	1.092 ± 1.917	n.s.	2.298 ± 2.37	2.345 ± 3.15	n.s.
Aromatic compounds	0.0103 ± 0.010	0.004 ± 0.002	**	0.005 ± 0.004	0.005 ± 0.003	n.s.
Nitriles	0	0.001 ± 0.005	n.a.	0	0.002 ± 0.005	n.a.
Non-acetate esters	35.14 ± 2.90	35.24 ± 6.56	n.s.	38.03 ± 2.43	22.30 ± 7.02	***
Oxirane	11.6 ± 2.55	2.594 ± 2.11	***	5.991 ± 2.30	2.608 ± 2.03	***
Sulphur compounds	0.265 ± 0.52	0.215 ± 0.159	n.s.	0.206 ± 0.37	0.026 ± 0.024	*
Terpenes	0.039 ± 0.025	0.010 ± 0.007	***	0.021 ± 0.016	0.008 ± 0.008	**
Total esters	85.36 ± 3.53	84.26 ± 5.26	n.s.	75.02 ± 2.12	85.02 ± 8.88	***
**Metabolites** ^**b**^						
Total sugars	49.69 ± 9.61	59.53 ± 16.15	*	56.10 ± 14.87	73.79 ± 26.88	*
Reducing sugars	14.33 ± 1.27	23.38 ± 4.04	***	20.08 ± 8.40	22.06 ± 4.61	n.s.
Sucrose	29.74 ± 8.26	44.97 ± 12.41	***	28.65 ± 7.25	53.38 ± 5.82	***
TBARS	4.04 ± 1.09	3.73 ± 0.79	n.s.	3.35 ± 1.35	3.80 ± 0.82	n.s.

^a^Mean relative % abundance of VOC class as a ratio of total VOC area across all samples.

^b^Mean abundance as mg/g FW.

### VOCs discriminated amongst time points and cut sizes

Despite the different patterns of VOCs across seasons, analysis with PerMANOVA revealed that within both the 2013 and 2014 cut size data sets, VOC profiles discriminated significantly amongst both days of storage (*P* < 0.0001; R^2^ = 0.531 for 2013 and *P* < 0.0001; R^2^ = 0.656 for 2014) and cut sizes (*P* < 0.01; R^2^ = 0.087 for 2013 and *P* < 0.05; R^2^ = 0.041). There was also a significant interaction between days and cut size in both years (*P* < 0.01; R^2^ = 0.154 for 2013 and *P* < 0.05; R^2^ = 0.101 for 2014). Overall the PerMANOVA analysis accounted for 77.2% of the variation in 2013 and 78.7% in 2014.

**Figure 1:**
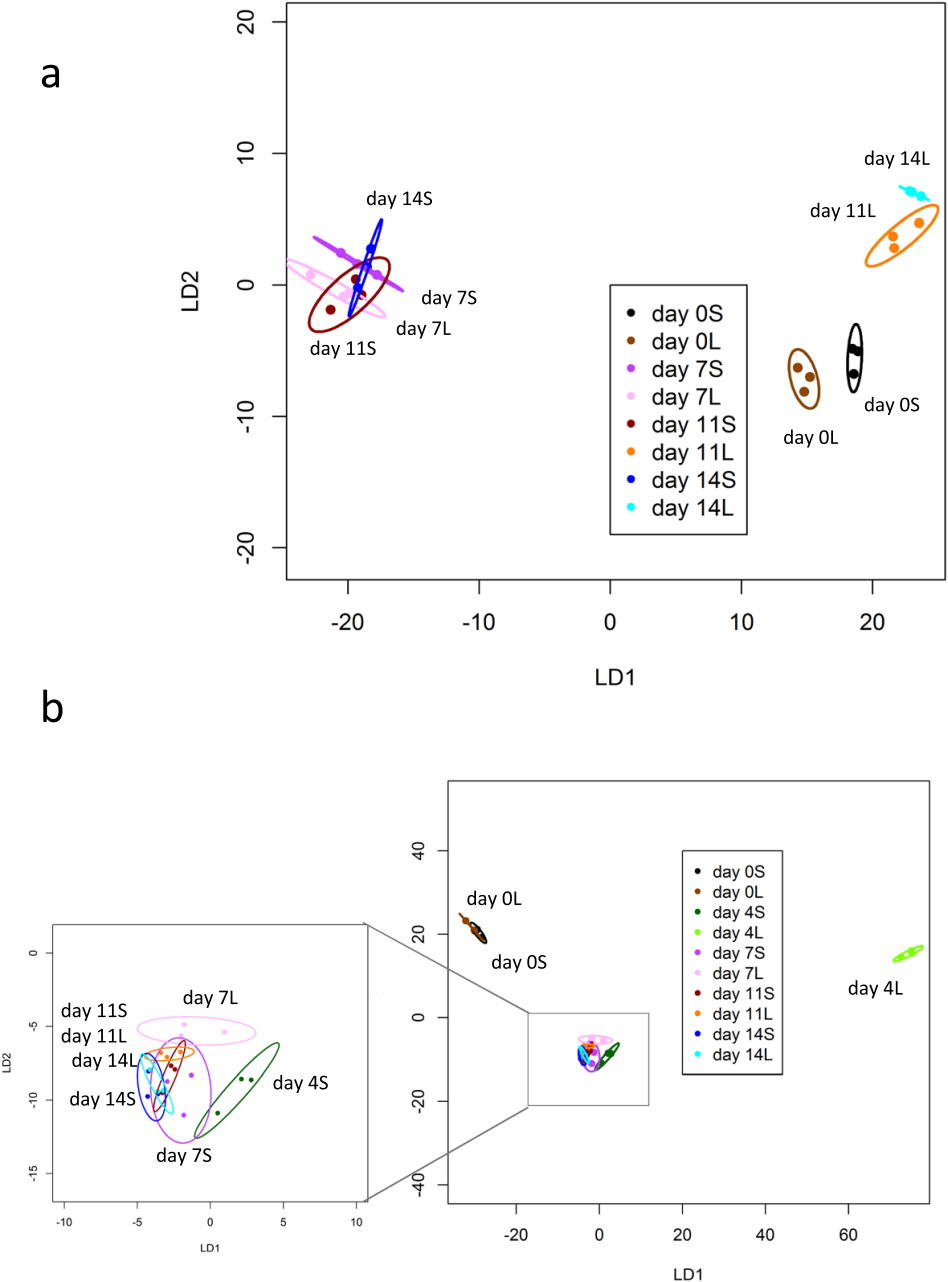
Canonical Analysis of Principal coordinates related to cut size and time of storage based on all melon VOCs using TD-GC-TOF-MS: combined time and cut size into a single sample category (**a**) 0, 7, 11, and 14 days for 2013 and (**b**) 0, 4, 7, 11, and 14 days for 2014. L is large cut size (3 × 2 cm); S is small cut size of melon cubes. (Each ellipse represents the 95% confidence interval. The plots use LD1 and LD2 with a percentage of correct classification of 66.6% and *P* < 0. 01 for (**a**) and 76.7% and *P* < 0.01 for (**b**); (n = 3).

Linear discrimination plots from CAP analysis separated days of storage with a correct classification of 83.3% for 2013 and 70% for 2014. When cut size was combined with days of storage, CAP discriminated amongst samples (*P* < 0.001) in both years, with a correct classification of 66.6% in 2013 and 50% in 2014. In both years the day 0 samples were well separated from the other time points, however in 2013 small and large cut size were well-separated at day 0 whereas in 2014 they were not. On day 7, 11 and 14 cut sizes were well separated in 2013 but not in 2014 (Fig. 1a and b; Supplementary Fig. S1).

Given that in both seasons it was possible to discriminate according to cut size and days of storage using the whole VOC profile, the data from the two seasons were combined to derive a dataset consisting of 44 VOCs that were detected in both years. PerMANOVA showed that discrimination across time of storage was retained in this reduced dataset (*P* < 0.0001; R^2^ = 0.214) however discrimination across cut size was lost. Notably the VOC data still discriminated profiles across the two seasons (*P* < 0.0001; R^2^ = 0.289). CAP analysis separated seasons and day 0 from the other time points but did not separate cut size (Supplementary Fig. S2).

### Temperature and time of storage affected VOC profiles in both seasons

**Figure 2:**
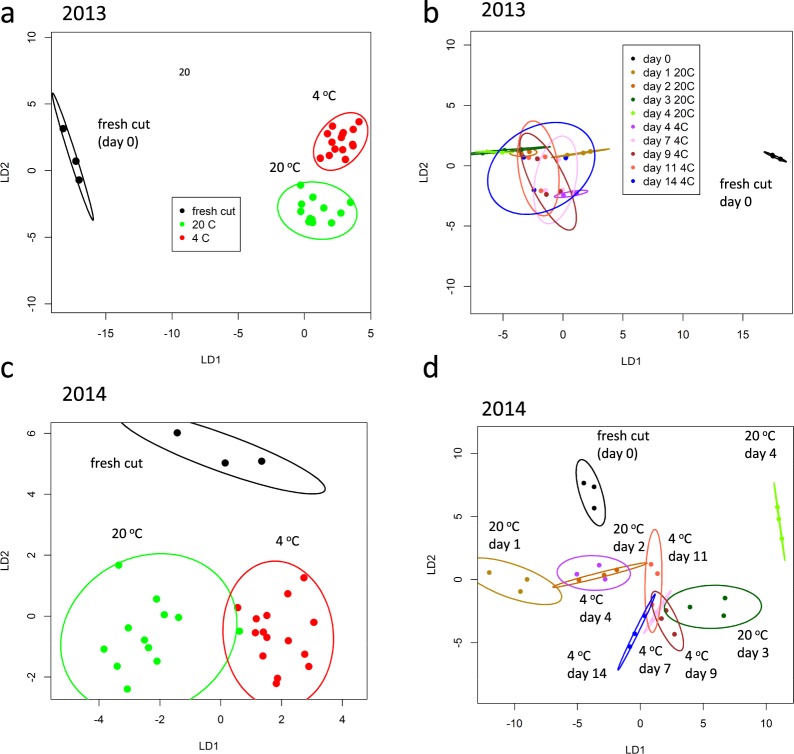
Canonical Analysis of Principal coordinates related to temperature and time of storage based on all VOCs from 2013 and 2014 melons using TD-GC-TOF-MS: (**a**) 2013 and (**c**) 2014, temperature of storage at 4 °C and 20 °C; (**b**) 2013 and (**d**) 2014 days and storage at 4 °C and 20 °C. Each ellipse represents the 95% confidence interval. The plots use LD1 and LD2 with a percentage of correct classification of (**a**) 63.3% (n = 3 for fresh, n = 12, for 20 °C, n = 15 for 4 °C (**b**) 50% and (n = 3); (**c**) 93.3% (n = 3 for fresh, n = 12, for 20 °C, n = 15 for 4 °C; (**d**) 56.7% (n = 3). Key for (**a**) applies to (**c**) and key for (**b**) applies to (**d**).

VOC profiles were able to discriminate between larger (3 × 2 cm) cut size samples held at the two contrasting temperatures of 4 °C and 20 °C using PerMANOVA, with *P* < 0.001; R^2^ = 0.571 for 2013 and *P* < 0.001; R^2^ = 0.339 for 2014. Overall, the PerMANOVA analysis accounted for 73% of the variation in 2013 and 63% in 2014. CAP analysis separated fresh cut (day 0) from stored samples in both seasons, and the two storage temperatures in 2013, but not 2014 (Fig. 2a,c). However, when individual temperature × time points are considered, CAP separation was much better amongst the 2014 samples (Fig. 2b,d). In 2014, VOCs clearly separated samples held at 20 °C for 0, 3 and 4 days while days 1 and 2 were clearly discriminated from the rest but not from each other. In 2013, day 1 was separated from day 2 at 20 °C, but day 2 was not separated from later time points (day 3 and day 4; Fig. 2b,d). At 4 °C, separation was not as good as at 20 °C in either year. In 2014, it was possible to discriminate day 4 from all other time points while VOC profiles from all other samples overlapped (except day 7 and day 14). In 2013, VOC profiles for all five samples overlapped each other at the 95% confidence interval.

**Figure 3:**
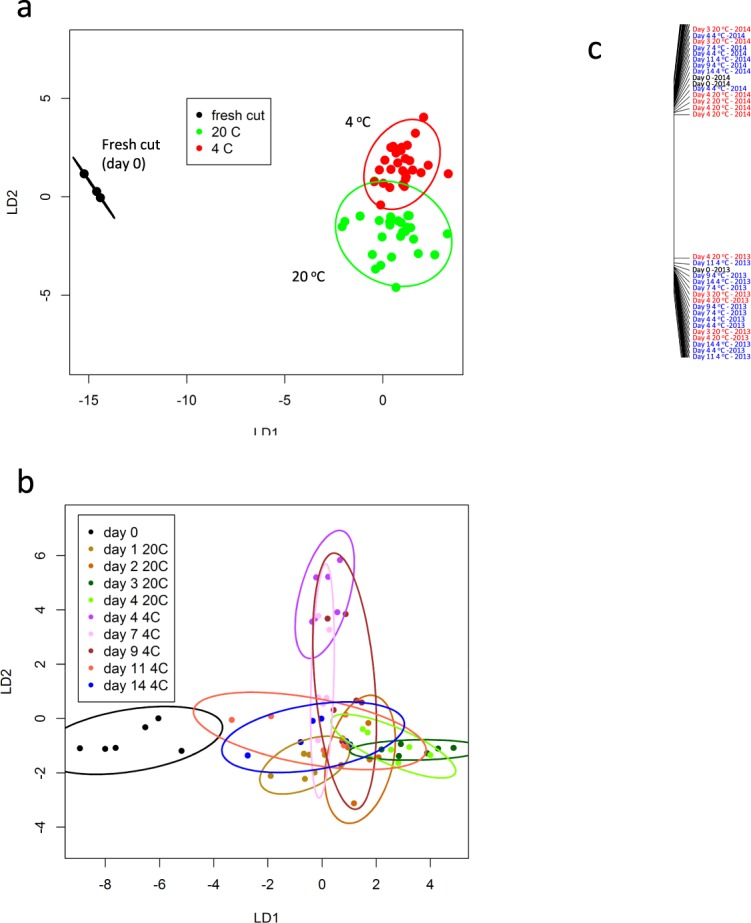
Canonical Analysis of Principal coordinates related to temperature and time of storage based on all VOCs combined from 2013 and 2014 melon using TD-GC-TOF-MS: A CAP model was produced for melon samples (**a**) temperature of storage at 4 °C and 20 °C, (**b**) days and storage at 4 °C and 20 °C into a single sample category and (**c**) season. Each ellipse represents the 95% confidence interval. The plots use LD1 and LD2 (**a**) with a percentage of correct classification of 96.7% (n = 3 for fresh, n = 12, for 20 °C, n = 15 for 4 °C), (**b**) with a percentage of correct classification = 48.3% (n = 3) and LD1 for (**c**) with a correct classification of 100% (n = 30).

When all data for both years was combined, (89 VOCs), PerMANOVA showed that it was still possible to discriminate by temperature of storage (*P* < 0.001; R^2^ = 0.092), and across storage times (*P* < 0.001; R^2^ = 0.12). CAP analysis showed that fresh cut (day 0) was still discriminated from the two storage temperatures (Fig. 3a). For individual samples, fresh cut was discriminated by CAP from all storage time points at 20 °C. However, although day 1 at 20 °C was discriminated from days 3 and 4, day 2 overlapped all three (Fig. 3b). At 4 °C day 4 was discriminated from days 11 and 14 but days 7 and 9 VOCs overlapped both day 4 and later time points (Fig. 3b). Thus, combined season data were more discriminatory amongst time points at 4 °C than data from 2013 and equivalent to separation for the 2014 data alone. In the combined data set, VOCs discriminated extremely well between the two seasons (*P* < 0.001; R^2^ = 0.664) indicating and reflecting significant differences in VOC production as described earlier (Fig. 3c).

### WCNA enabled identification of VOC subsets with potential as markers for assessing time × temperature of storage that are independent of cut size

Weighted Correlation Network Analysis (WCNA) on the 41 shared VOCs between the two seasons of the temperature experiment, identified two modules which were least significant for season and while being significant for time and day of storage (yellow and grey modules, Supplementary Fig. S3). Modules represent groups of VOCs with a similar pattern of change in relation to the trait, in this case temperature or days of storage. Eleven VOCs from these two modules varied significantly with temperature and/or time of storage (*P* < 0.05; Table 2). Analysis of these profiles with PerMANOVA showed a reduction of variability by season to 2.5% (F_1,59_ = 2.75, n.s.) whilst temperature and time of storage accounted for 36% (F_2,59_ = 19.9, *P* < 0.001) and 14% (F_7,59_ = 2.16 *P* < 0.05) respectively. This indicates that they retain most of the discriminatory power of the full data set suggesting their possible application as markers for shelf life prediction. Of these eleven VOCs, five ((methyldisulfanyl)methane, methyl propanoate, methyl thioacetate, isobutyl propionate and thiopivalic acid) fell significantly from day 0 to the first storage time point at both storage temperatures in both years: indicating that these may serve as useful markers for the early time points after processing.

**Table 2:** Subset of 12 volatile organic compounds (VOCs) identified by Weighted Correlation Network Analysis (WCNA) from the set of 41 common compounds shared between 2013 and 2014 seasons of the two temperature experiment.

VOC Name (IUPAC)	NIST name	Class
1-Isopropyl-2-methoxybenzene^b^	o-Isopropylanisole	Aromatic
2-Methylpropanoic anhydride^b^	Propanoic acid, 2-methyl-, anhydride	Acid anhydride
2-Pentanyl propionate	2-Pentanol, propanoate	Non-acetate Ester
Acetic acid, 2-phenylethyl ester^b^	Acetic acid, 2-phenylethyl ester	Acetate Ester
(Methyldisulfanyl)methane^a^	Disulfide, dimethyl	Sulphur
Isobutyl propionate^a,b,c^	Propanoic acid, 2-methylpropyl ester	Non-acetate Ester
Methyl propanoate^a,b,c,^	Methyl propionate	Non-acetate Ester
Methyl thioacetate^a,b,c^	Methyl thiolacetate	Sulphur
N-Acetoxy-N-acetylacetamide	N,N,O-Triacetylhydroxylamine	Amine
Propyl 2-methyl-butanoate^b,c^	Butanoic acid, 2-methyl-, propyl ester	Non-acetate Ester
Thiopivalic acid^a^	Thiopivalic acid	Sulphur compound

^a^Five VOCs whose abundance falls significantly between day 0 and the first storage time point at both storage temperatures in both years.

^b^Seven VOCs also present in the 44 VOCs shared between the two seasons of the cut size experiment.

^c^Four VOCs that are not correlated with cut size across both seasons.

Of the eleven VOCs identified as potential markers (Table 2), seven were also amongst the 44 VOCs shared between the two seasons in the cut size experiment. Of these seven VOCs, the relative abundance of four (isobutyl propionate, methyl propanoate, methyl thioacetate and propyl 2-methyl-butanoate) was not significantly correlated with cut size when analysed by WCNA (P < 0.05, Table 2), indicating that they could be useful markers for time × temperature changes independently of cut size. Thus, isobutyl propionate and methyl propanoate could be useful markers for early changes in quality irrespective of cut size.

### Storage time but not cut size affected abundance of esters and expression of ester biosynthetic genes

**Figure 4:**
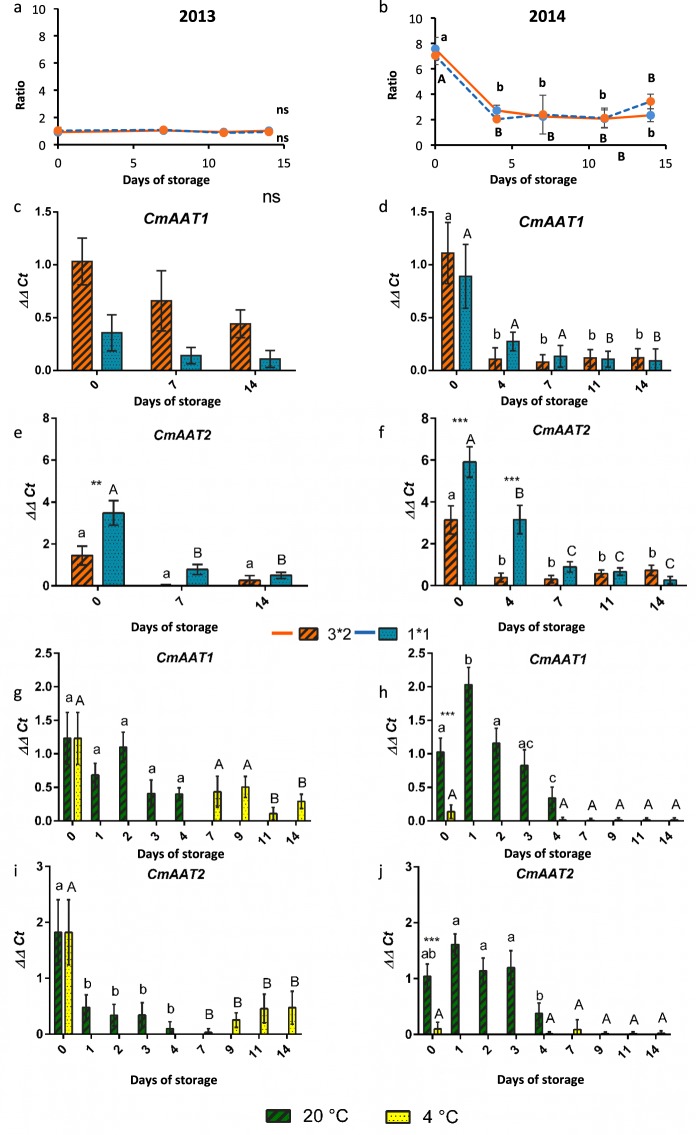
Changes in gene expression of genes related to ester biosynthesis over storage time and temperature, cut size, and across the two seasons. (**a**,**c**,**e**,**g**,**i**) 2013 and (**b**,**d**,**f**,**h**,**j**) 2014. (**a**–**d**) Compare cut size (small and large) (**e**–**h**) compare storage at two temperatures (4 °C and 20 °C); (**a**,**b**) ratio of acetate to non-acetate esters in total VOC profile for each time point; (**c**,**d**,**g**,**h**) expression of alcohol acetate transferase (*AAT1*), (e,f,i,j) expression of alcohol acetate transferase (*AAT2*); n = 3 bars indicate ± SE. Asterisks denote significant differences between the two cut sizes at each time point: *P < 0.05, **P < 0.01, ***P < 0.001; different letters above bars indicate differences across time points (uppercase for large 3 × 2 cm (L) and lower case for small 1 × 1 cm (S) cut sizes); ns indicates that there were no significant differences across any of the time points or cut sizes.

In the 2013 cut size experiment, the abundance ratio between the two major groups of esters (acetate to non-acetate) was constant throughout the storage period, starting at 1.06 ± 0.07 and 1.17 ± 0.22 respectively for the larger and smaller cut sizes (Fig. 4a). In contrast, in 2014, there was a significant reduction in the abundance ratio between acetate and non-acetate esters in the first 4 days of storage in both cut sizes (*P* < 0.01), however, there were no significant differences between the two cut sizes (Fig. 4b). The difference in the change in ester ratio between the two seasons was mirrored by the pattern of *CmAAT1* gene expression (Fig. 4c and d). In 2013, there were no significant differences in expression across time points in *CmAAT1* expression, whereas in 2014 expression dropped off significantly by day 4 in the larger cut size and by day 11 in the smaller cut size compared to the day 0 expression. In contrast, *CmAAT2* expression fell over time in the smaller cut size in both seasons, although in the larger cut size, expression only fell significantly over time in 2014 but not in 2013 (Fig. 4e,f). There were no significant differences between the two cut sizes in *CmAAT1* expression. However, expression of *CmAAT2* differed in expression between cut sizes at early time points in both seasons, showing a higher expression in the smaller cut size.

### Weighted Correlation Network Analysis (WCNA) of the 2014 cut size dataset identified two esters whose change in abundance correlates with the change in *CmAAT1* gene expression during storage

**Figure 5:**
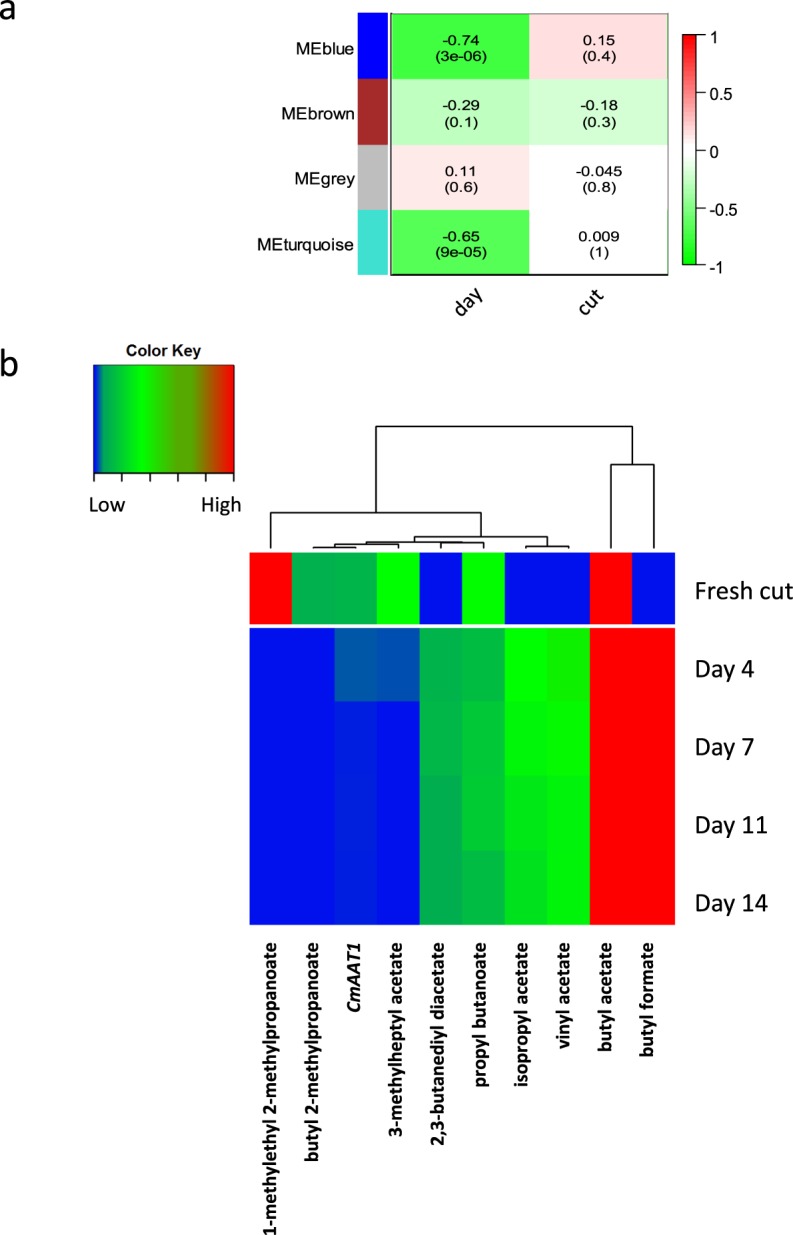
Multi-trait correlation analysis of all ester VOCs and gene expression of genes related to ester biosynthesis, *CmAAT1* and *CmAAT2* during the 2014 cut size experiment: (**a**) WCNA modules where score and significance (as *P* values in brackets) in relation to the traits (days of storage and cut size) are from a Pearson analysis based on WCNA soft-thresholding power = 8 and Module size = 4; (**b**) Multi-trait analysis heat map and hierarchical clustering based on day of storage using VOCs grouped into the Turquoise module in (**a**) and which were significantly correlated with days of storage. Blue indicates low, green intermediate and red high abundance of the character (transcript or VOC).

WCNA was used for a multitrait analysis of correlations amongst changes in shared VOCs between 2013 and 2014 seasons, metabolites in 2014, and gene expression in 2014. This analysis did not reveal any strong patterns of correlation (Supplementary Fig. S4), apart from a correlation in the increase of reducing sugars with ethyl propanoate and methyl 2-methyl-butanoate. However, applying WCNA to further investigate correlations between changes in ester VOCs and changes in expression of the two *CmAAT* genes revealed more interesting results. The 43 esters that were detected in the cut size 2014 experiment, combined with the expression of *CmAAT1* and *CmAAT2* real time PCR data, fell into four modules (blue, brown, grey and turquoise) using WCNA in a multi-trait analysis, based on their change in abundance/expression over time, or across the two cut sizes (Fig. 5a). Expression of *CmAAT2* grouped with 24 VOCs in the grey module, which was not correlated significantly with either days of storage or cut size. Expression of *CmAAT1* clustered with ten esters in the turquoise module, which was negatively correlated to time of storage (*P* < 0.001). Nine of these esters showed a significant correlation with the days of storage (*P* < 0.05). These were clustered hierarchically and are displayed as a heat map (Fig. 5b). Two VOCs showed a close correlation with the *CmAAT1* gene expression: butyl 2-methylpropanoate and 3-methylheptyl acetate indicating a possible role for *Cm*AAT1 in their biosynthesis.

### Storage at higher temperature for a shorter time abolished the change in ester ratios but still resulted in a loss of *CmAAT* gene expression

Storage of the fresh cut melon at 20 °C for 4 days did not result in a reduction of acetate: non-acetate ester ratio in either season (Supplementary Fig. S5). However, storage at 20 °C resulted in a reduction in the expression of both *CmAAT1* and *CmAAT2* in both seasons, although the fall in *CmAAT1* expression in the 2013 season was not statistically significant (Fig. 4g–j).

### Expression of ERF and TINY transcription factors was affected by cut size, season and post-harvest storage time

**Figure 6:**
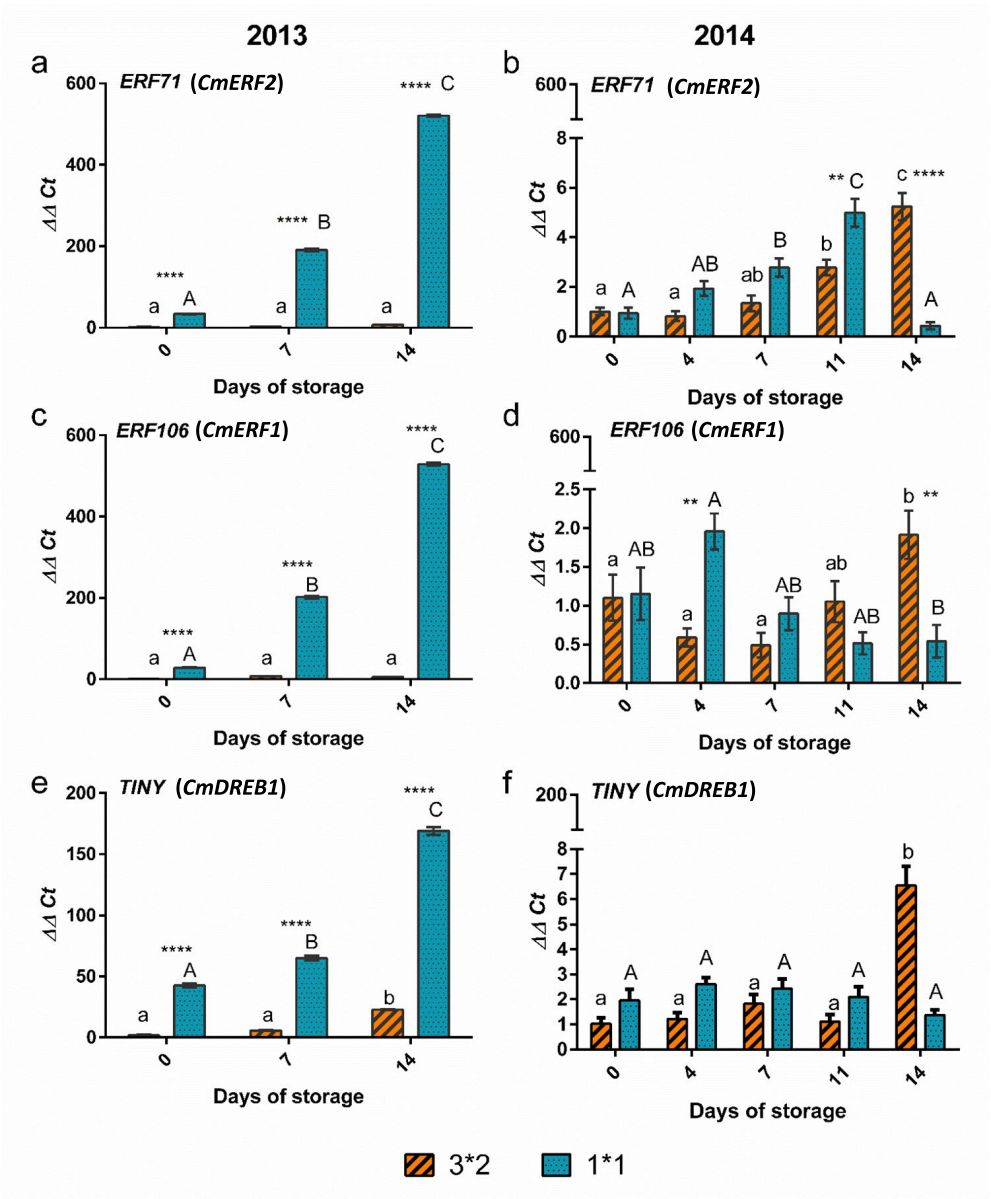
Changes in gene expression of *ERF* and *TINY* transcription factors over storage time with cut size and across the two seasons. *ERF71* (**a**) 2013 (**b**) 2014; *ERF106* (**c**) 2013 (**d**) 2014; *TINY (DREB)* (**e**) 2013, (**f**) 2014. n = 3 bars indicate ± SE. Different letters indicate statistically significant differences among different days of storage (*P* < 0.05). Asterisks denote significant differences between the two cut sizes (large, L and small, S): *P < 0.05, **P < 0.01, ***P < 0.001.

Expression of the melon *ERF71* (*CmERF2*) gene was dramatically up-regulated in the smaller cut size melon pieces during storage, with an increase in expression in both seasons (Fig. 6a,b): up to day 14 in 2013 and up to day 11 in 2014. In the 2014 season, this gene was also upregulated during storage in the larger melon pieces, increasing in expression throughout the storage period, however this was not the case in the 2013 season.

Expression of another AP2/ERF transcription factor, the melon *ERF106* (*CmERF1*) gene followed a very similar expression pattern to *ERF71* in the 2013 season but in 2014 the expression pattern was less consistent (Fig. 6c and d). There was an upregulation in later storage time points in the larger cut size, but there was no significant change in expression during storage in the smaller melon pieces.

Expression of the melon *TINY* gene (*CmDREB1*) followed a very similar pattern to the two *AP2/ERF* genes in 2013, increasing in expression in both melon cut sizes, however, in 2014 the change in expression was greatly reduced and only the larger cut size was significantly more highly expressed at day 14 than day 0 (Fig. 6e,f). Notably, the expression of all three transcription factors in the small cut size melon pieces in 2013 was dramatically greater than in 2014.

**Figure 7:**
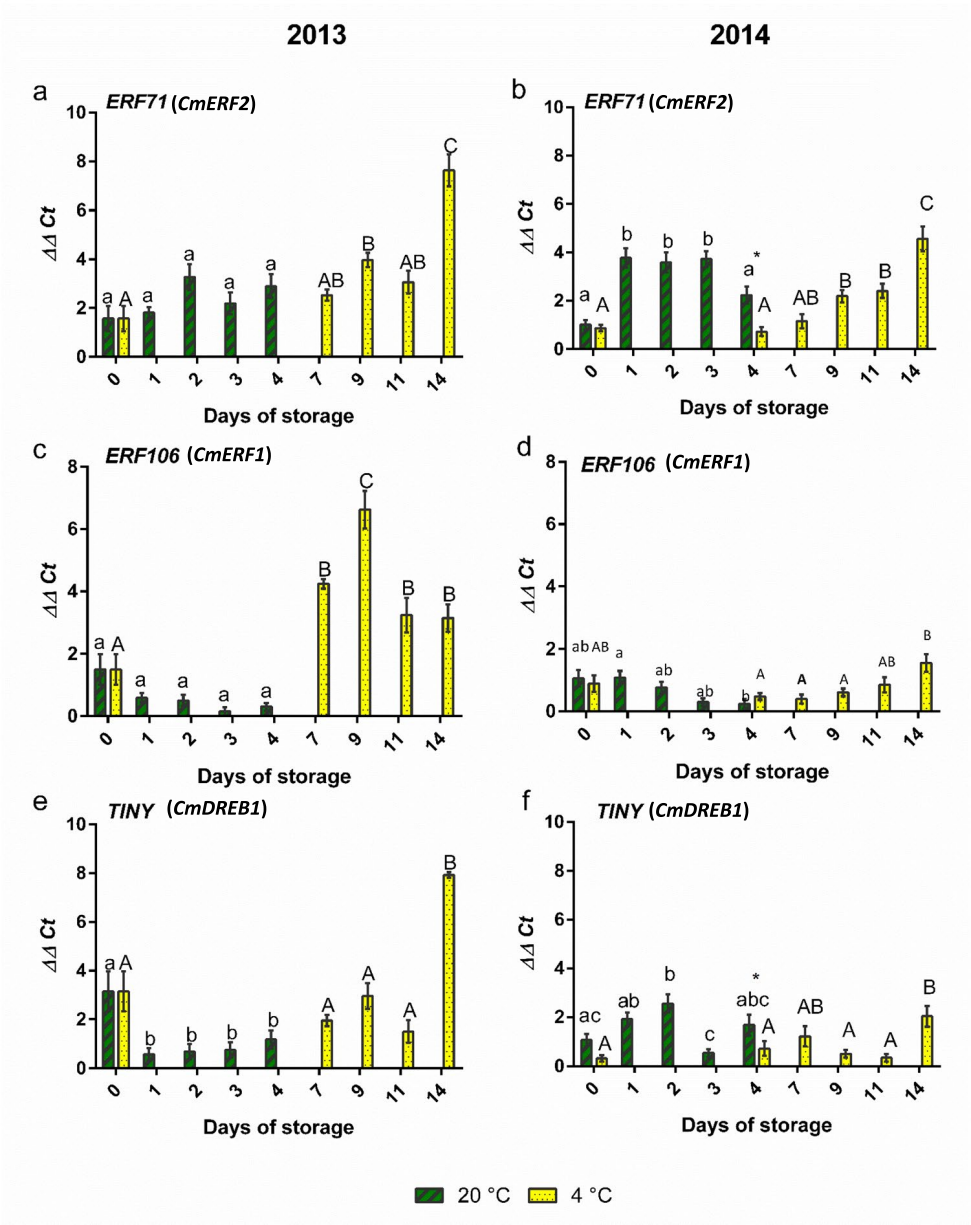
Changes in gene expression of *ERF* and *TINY* transcription factors over storage time with storage temperature and across the two seasons. *ERF71* (**a**) 2013 (**b**) 2014; *ERF106* (**c**) 2013 (**d**) 2014; *TINY* (*DREB*) (**e**) 2013, (**f**) 2014. n = 3; bars indicate ± SE. Different letters indicate statistically significant differences among different days of storage (*P* < 0.05). Asterisks denote significant differences between two temperatures: *P < 0.05, **P < 0.01, ***P < 0.001.

Storage of the fresh cut melon at 20 °C instead of 4 °C for a shorter period did not have a dramatic effect on expression of these genes (Fig. 7). *ERF71* (*CmERF2*) expression was not significantly affected by storage at either temperature in 2013. In the 2014 melon pieces, there was a rise in expression after the first day of storage at 20 °C but no further rise in the following days of storage (Fig. 7a,b). *ERF106* (*CmERF1*) was expressed at higher levels when melon pieces were stored at 4 °C in the 2013 season but in 2014 the difference was less evident (Fig. 7c,d). *TINY* (*CmDREB1*) expression also appeared to be higher at 4 °C in the 2013 season but again this difference between the two storage regimes was not seen in the 2014 season (Fig. 7e,f).

### Sugar content was affected by season, cut size and storage regime, and oxidative stress was greater in the larger cut size

There were differences in the mean sugar content of the fruit across all the sampling times between the two seasons, in both cut size and in the storage temperature experiments. All sugars were less abundant in 2013 in the temperature experiment (Table 1), while in the cut size experiment, only the content of total sugars and sucrose was significantly lower in 2013.

**Figure 8:**
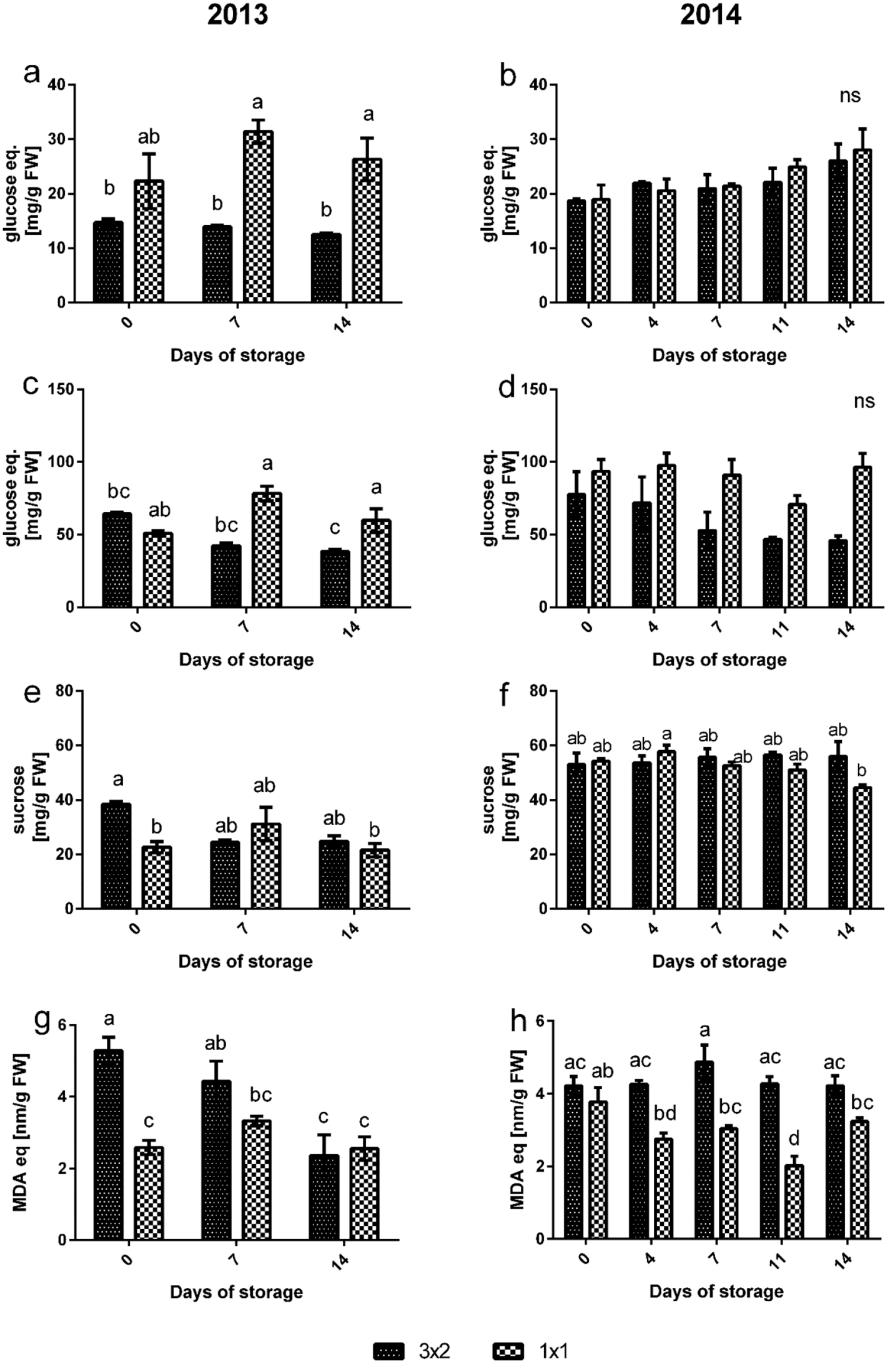
Changes in sugars and oxidative stress over storage time with cut size and across the two seasons. Reducing sugars (**a**) 2013 (**b**) 2014; total sugars (**c**) 2013 (**d**) 2014; sucrose (**e**) 2013 (**f**) 2014; TBARS expressed as MDA equivalents (**g**) 2013 (**h**) 2014 (large, L and small, S). n = 3; bars indicate ± SE. Different letters indicate statistically significant differences (*P *< 0.05).

In 2013, amounts of both reducing sugars and total sugars were significantly higher in the smaller cut size samples than in the larger cut size after storage for a week, and the difference was retained even after 2 weeks of storage (Fig. 8a,c). A similar trend was seen in 2014 for reducing sugars after 11 and 14 days (Fig. 8b) and for total sugars at all time-points during storage (Fig. 8d), however the differences were not statistically significant. In both years, total sugar and reducing sugar content was stable through the storage period within each cut size group. Overall, sucrose content was significantly higher (*P* < 0.0001) in 2014 compared to 2013 (Fig. 8e,f), but there were no consistent differences between the two cut sizes.

The TBARS assay indicated an increased oxidative stress in the larger melon pieces which was consistent across most time points and in both seasons (Fig. 8g,h). However, there was no significant difference in TBARS across the two seasons (Table 1).

The temperature regime did not have a dramatic effect on retention of sugars during storage (Supplementary Fig. S6). The greatest effect was on retention of sucrose, which was higher at 4 °C in the 2014 season than during the shorter period of storage at 20 °C, although this was not the case in 2013.

## Discussion

A similar total number and range of VOCs was detected compared to other studies^2,7^ across the whole sample set. In fact, a further 17 VOCs were detected here in *Cucumis melo* L. var. reticulatus cv Macigno, compared to the 82 detected by TD-GC-ToF-MS from *C*. *melo* var. cantalupensis cv. Arapaho. This probably indicates differences amongst cultivars as has been previously noted^20,28^. Several compounds identified as “characteristic impact flavor and aroma compounds” (CIFAC) in *Cucumis melo*^29–36^ were recovered including 2-methylbutyl acetate, hexyl acetate, 3-hexenyl acetate, ethyl 2-methylbutanoate and eucalyptol.

The differences in abundance of several classes of VOCs between the 2013 and 2014 data sets as a whole suggest that the development of aroma differed considerably in the two seasons. Melons were harvested at the same stage of maturity from the same location, at the same time of year (June-July) and grown using the same standard agronomical management. Therefore, the differences are likely to be due to the physiological status of the fruit at maturity, dependent on the seasonal meteorological variation. This is supported by the significantly higher sugar content in the melons in 2014 compared to 2013. Even under greenhouse conditions, seasonal effects have been noted in relation to accumulated sucrose^37^, the main sugar that accumulates in melon fruits. When melons were grown in the summer compared to the spring, they accumulated less sugars, this may be due to a higher temperature accelerating plant growth and fruit development, but providing less time to load sugar. Weather data for 2013 and 2014 near Mantova, Italy, where the melons were grown, show similar mean temperatures (Supplementary Table S2), although the 2013 season was slightly warmer, which would fit with the model of faster ripening fruit with less sugar accumulation.

The consistent mean abundance of all esters between 2013 and 2014 in the temperature experiment is in agreement with a previous study that compared aroma across seasons^15^. In this previous study, both the abundance of acetate and non-acetate esters was significantly different across years but total ester abundance remained stable. The difference in abundance of total esters in the cut size experiment contrasts with the Beaulieu^15^ report, although in the cut size experiment reported here, each class of ester VOCs (acetate and non-acetate) did differ significantly between the two years of the study. Aldehyde abundance was also season-independent in the Beaulieu^15^ study and was not significantly different between the two seasons in the cut size experiment here. However, here there was a significant change in abundance of aldehydes in the storage temperature experiment as well as a difference in composition: the only aldehyde detected in 2013 was (2E)-2-methyl-2-butenal, whereas in 2014 only methylpropenal was detected. Overall, there was more similarity in the seasonal effect on VOC class abundance between the cut size experiment and the Beaulieu study^15^, which was also conducted at 4 °C. This suggests that at a higher post-harvest storage temperature, seasonal effects on VOC content become more important.

The separation between cut sizes based on VOCs is consistent with a previous report on the effects of cut size on melon VOCs^38^ recorded after 15 days storage at 4 °C. However, results here show that the discrimination is affected by time of storage and season. VOC changes are important in that they may reflect changes in nutritional or sensorial value of the fresh cut fruit^7^. Hence, the inference is that cut size may affect quality within the time frame of commercial shelf life. These changes are most likely associated with the difference in wounding and or surface area to volume ratio, which in this experiment was 2.25 fold different between the two cut sizes. This is at least partly supported by the difference in expression of stress-related transcription factors, *ERF71 (CmERF2)*, *ERF106 (CmERF1)* and *TINY (CmDREB1)*, which in 2013 were much more highly expressed in the more wounded (small cut size) melon pieces. Previously, *CmERF1* and *CmERF2* were also up-regulated by abiotic stresses (drought and cold respectively), and *CmDREB1* by jasmonic acid in leaves, although effects of wounding were not tested^23^. However, the pattern of expression seen here was also very clearly affected by seasonality, indicating that factors other than wounding were regulating the expression of these genes as well as affecting the VOC profiles. In fact, *CmERF2* is indeed also up-regulated during fruit ripening^22^, thus the physiological state of the fruit in the different seasons may have affected the expression of this gene.

Surprisingly, the larger cut size generally elicited higher lipid peroxidation levels than the smaller cut size despite the greater wound damage to the smaller cut size. Moreover, there was no consistent increase in MDA over time of storage in either cut size. This contrasts with previous data^13,39^ showing maximal MDA levels in fresh cut flesh of a range of melon cultivars after 10–14 days of storage. The difference between cut sizes could be due to a greater activation of ROS scavenging enzymes, also known to be activated by wounding^13^. The lack of increase in lipid peroxidation with time may be due to the already high levels of MDA seen here, suggesting that near maximal levels of lipid peroxidation may already have been reached during or shortly after cutting.

In both years of the cut size experiments VOC profiles were better at discriminating times of storage amongst the large cut size than the small cut size, at least over the first 11 days of storage. This might reflect a more rapid deterioration of the smaller cut sized pieces. This is supported by data from Lamikanra and Richard^10^, where a very rapid loss of esters was found using very thin slices of melon flesh stored at 4 °C. However, it was not possible to discriminate between the two cut sizes using only VOCs shared between the two seasons. This indicates that it may be difficult to obtain markers specific for cut size that are not affected by the season.

The rapid fall in acetate:non acetate ester ratio over the first 4 days of storage in 2014 (but not in 2013) at 4 °C is in agreement with a previous report^16^, but was not seen in other experiments^7^. This suggests that pre-harvest factors may influence this change. However, ester ratios (acetate to non-acetate) remained very similar in the two cut sizes, indicating that cut size is not a factor influencing the change in ratio. The high expression of both *CmAAT1* and *CmAAT2* in the fresh cut melon is consistent with the high expression of these genes seen in ripe melon fruit and their activity in the production of several acetate and non–actetate esters^26^ also found here, including benzyl acetate, 2-phenylethyl acetate and 1-butyl propanoate. Expression of both genes fell significantly with storage time indicating a reduction in ester biosynthesis. Expression of *CmAAT1* was slightly, but not significantly, lower in the smaller cut size, perhaps related to increased damage to the tissue. The consistently greater expression of *CmAAT2* in the smaller cut size at earlier time points indicates that expression of this gene might be activated by wounding. Two ester products potentially linked to the activity of *CmAAT2*: ethyl heptanoate and ethyl octanoate^27^ were found amongst the VOCs common to both seasons in the cut size data, although their abundance did not correlate with the *CmAAT2* expression in relation to cut size. Moreover, the fall in *CmAAT1* gene expression with time of storage, did not correlate with a reduction in the abundance of any of the six putative CmAAT1 products reported by El-Sharkawy *et al*.^26^ (ethyl acetate, benzyl acetate, 2-phenylethyl acetate, ethyl propanoate, 1-butyl propanoate, ethyl 2-methylpropanoate and ethyl hexanoate), although these VOCs were present in the 2014 cut VOC profile. This suggests that *CmAAT1* may be contributing to the biosynthesis of other esters. Neither of the two VOCs whose change in abundance correlated with *CmAAT1* expression were identified by Freilich *et al*.^27^ as correlated with *CmAAT1* expression in their RIL populations, or were identified by El Sharkawy^26^ as products of CmAAT1 enzyme activity. However, it is possible that the different cultivars used or the changes in metabolic status induced by the post-harvest storage conditions might provide alternative substrates for the CmAAT enzymes or that their activity towards different substrates might be affected. Further analysis of CmAAT1 and CmAAT2 activities on potential alcohol substrates for the synthesis of these esters would need to be carried out.

Temperature of storage is known to have a strong effect on fresh cut melon shelf life^40^ and VOC profiles^7,15^. Here results indicated however, that the discrimination amongst storage temperatures is affected by the season. Furthermore, it was possible to identify a sub-set of eleven common VOCs across seasons that retained discrimination according to temperature of storage. These include five VOCs that seem to be associated with early time points after processing, and two of these that were also independent of cut size. These may be useful as potential markers for assessing time after processing.

In conclusion, results indicate that year on year climate variation has a complex effect on the physiological status of melon flesh and the retention of aroma in fresh cut melon during storage. This indicates the importance for the industry of assessing the optimal harvest time based not only on external appearance but also on a combination of physiological, molecular and biochemical markers.

## Materials and Methods

### Plant material, postharvest storage conditions and sample preparation

Melon (*Cucumis melo* L. var. reticulatus cv Macigno) fruits were field grown in Mantova (Italy) by the same commercial grower in 2013 and 2014. Over 60 fruits were harvested at the optimal commercial stage when an abscission circle was observed on the fruit (3/4 slip stage). The uniformity of ripening at harvest was assayed by measuring the level of chlorophyll degradation in the fruits with a non-destructive portable device (DA-meter)^41^. After harvest whole fruits were transferred to the laboratory within 3 hours, washed in a sodium hypochlorite solution (100 ppm), then mesocarp and endocarp were removed and discarded. The mesocarp (pulp) was cut in cube-shaped portions, using a sharp knife, to either 1 × 1 cm (small) or 3 × 2 cm (large). Both sizes were used for the cut size experiments and only the larger cubes for the two-temperature experiments. About 170 g of fresh-cut melon pieces were randomly selected from the mixed sample from the over 60 fruits. They were enclosed in plastic “clam shell” boxes (20 × 16.5 × 5 cm) secured with food grade low density poly ethylene plastic film to ensure the fruit material was protected from external microbial contaminants during the experiment. Boxes were stored at 4 °C in the dark (cut size experiments) and 20 °C or 4 °C in the dark for the two-temperature experiments. Sampling was performed in triplicate after 7, 11 and 14 days of storage (2013) and after 4, 7, 11 and 14 days (2014) for the cut size experiment. For the two-temperature experiment sampling was every day at 20 °C (day 1, 2, 3, 4) and every two days after one week at 4 °C (day 7, 9, 11, 14). Control samples were collected at harvest (day 0). Samples were frozen in liquid nitrogen and stored at −80 °C.

### VOC Sampling with TD tubes and analysis

To sample the VOCs, sealed boxes containing the melon pieces were transferred to 20 °C for 1 h to equilibrate. Then, an Easy VOC pump (Markes international Ltd) was used to pass a volume of 200 mL head-space through SafeLok tubes (Markes international Ltd) packed with Tenax TA and SulfiCarb sorbents, by inserting a needle into the box. Three biological replicates were performed for each sample. Samples were collected at the University of Milano and the tubes transported to Cardiff University for analysis.

A TD100 (Markes international Ltd) was used to desorb the tubes in the trap with the following conditions: desorption for 10 min at 280 °C with a trap flow of 40 mL/min. Desorption of trap at a rate of 40 °C/s to 300 °C with a split ratio of 11:1 into the GC (7890 A; Agilent Technologies, Inc). VOCs were separated over 60 m, 0.32 mm ID, 0.5 μm film thickness Rxi-5ms (Restek) using the following temperature program: 5 min at 35 °C initially, 5 °C/min to 100 °C followed by 15 °C to 250 °C and a final hold of 5 min (total run time 33 min). The BenchTOF-dx mass spectrometer (Almsco International) was operated at  an ion source temperature of 275 °C, ions were collected in a mass range of 30 to 350 m/z. A retention time standard (C8-C20, Sigma Aldrich) was prepared by injection of 1 μL of the standard mixture directly onto a collection tube (Tenax TA, Sulficarb) and analysed under the same conditions as the samples.

GC-MS data were processed using MSD ChemStation software (E.02.01.1177; Agilent Technologies, Inc), and deconvoluted and integrated with AMDIS (NIST 2011) after first constructing a retention-indexed custom MS library. MS spectra from deconvolution were searched against the NIST 2011 library^42^ (version 2.0 g) and only compounds scoring >80% in forward and backward fit were included. Putative identifications were based on match of mass spectra (>80%) and retention index (RI +/− 15).

### Analysis of sucrose, reducing sugars and total sugars

For the determination of sucrose, 2 g of tissue was extracted by homogenization in a mortar with 20 mL of water. The 2 g sample was a sub-sample from 170 g of melon flesh for each sample derived from a total of five melon fruit. The mixture was then centrifuged at 4000 × g for 20 min. Sucrose assay was performed by mixing 0.2 mL of extract with 0.2 mL of 2 N NaOH and incubated in a water bath at 100 °C for 10 min, then 1.5 mL hot resorcinol buffer (containing 30% hydrochloric acid, 1.2 mM resorcinol, 4.1 mM thiourea 1.5 M acetic acid) was added to samples and incubated in a water bath at 80 °C for other 10 min. After cooling to room temperature, the optical density was determined spectrophotometrically at 500 nm, using a sucrose standard curve (0, 0.5, 1, 1.5 and 2 mM).

Reducing sugars were analysed spectrophotometrically by adding 0.2 mL of a 1.52 M potassium sodium tartrate solution containing 62.6 mM dinitrosalicylic acid (DNS) to 0.2 mL of extract. The reaction mixture was heated to 100 °C for 5 min, 1.5 mL of distilled water were added and absorbance readings were taken at 530 nm. The reducing sugars were expressed as glucose equivalent using a glucose standard curve (0, 1, 2, 3 and 4 mM).

Total sugars were determined spectrophotometrically from the same extract, following the anthrone method^43^ with slight modifications. The anthrone reagent (10.3 mM) was prepared dissolving anthrone in ice-cold 95% H_2_SO_4_. The reagent was left to stand for 30–40 min before use, 0.5 mL extract was placed on top of 2.5 mL of anthrone reagent, incubated in ice for 5 min and then vortexed vigorously. The reactions were heated to 95 °C for 10 min and left to cool in ice. Readings were performed at 620 nm. Calibration curve was carried out using a glucose standard solution.

### Thiobarbituric Reactive Substances (TBARS) Assay

Lipid peroxidation was determined using the thiobarbituric acid reactive substances (TBARS) method^44^. 1 g of tissue was homogenized in 5 mL of trichloroacetic acid (TCA) 0.1% w/v and centrifuged at 4500 × g for 10 min. 1 mL of the supernatant was mixed with 4 mL of 20% (w/v) TCA, 25 μL of 0.5% thiobarbituric acid (TBA) and dH_2_O. After vortexing, the mixture was heated to 95 °C (30 min) in a water bath and cooled on ice. Absorbance at 600 nm was subtracted from the absorbance at 532 nm (as an index of non-specific turbidity) and the concentration of TBARS was expressed as malondialdehyde (MDA) equivalents (nmol g^−1^ F.W.), calculated using the Lambert-Beer law with an extinction coefficient ε = 155 mM^−1^ cm^−1^.

### Total RNA isolation and qRT-PCR gene expression analyses

Total RNA was extracted from 100 mg of tissue using the Spectrum Plant Total RNA Kit with on-column DNase-treatment (Sigma-Aldrich, Italy) according to manufacturer’s instructions. RNA concentration was assessed using a NanoDrop N-1000 spectrophotometer (NanoDrop technologies). Total RNA (5 µg) was reverse transcribed using Superscript III reverse transcriptase (Invitrogen, Italy) and a mix of random primers and oligo-dT (10 µM). The first strand synthesis reaction was incubated at 42 °C for 1 h. In order to avoid genomic DNA amplification, total RNA was treated with DNase I (Invitrogen, Italy). Specific primers (Supplementary Table S3) for expression analyses were designed based on the sequences found in the melon EST database (http://cucurbitgenomics.org/est/melon). SYBR green chemistry was used for real-time PCR gene expression analyses using an ABI 7300 (Applied Biosystems) machine. The amplification program was: 1 cycle at 50 °C for 2 min then at 95 °C for 2 min; 40 cycles at 95 °C for 30 s; 55 °C for 1 min and 72 °C for 30 s (signal acquisition stage); 72 °C, 10 min and dissociation curve to check the absence of primer dimers and other amplification by-products. Actin was used as the reference gene (Supplementary Table S3) and the expression was calculated with the ΔΔCt method.

### Statistical Analysis

VOC data were analysed using R software, PerMANOVA and CAP analysis (Adonis function in the R package “Vegan” followed by function CAPdiscrim in the R package “BiodiversityR”) performed as described by Anderson & Willis^45^. Linear discriminant plots were produced for storage day and temperature and a 95% confidence interval was fitted. Sub-sets of compounds correlated with the treatment: day and/or temperature were identified using Weighted Correlation Network Analysis a scale-free topology criterion method (WCNA package in R^46^). WCNA groups characters with similar patterns of change with relation to a trait (in this case cut size, season or temperature) into “modules”. These are associated with a Pearson’s coefficient of probability and the significance of this correlation. Soft threshold power (which determines the threshold for similarity) was selected as indicated in text and figures. Module size used was = 3 (this sets the size of minimum size of the modules). Hierarchical cluster analysis (HCA) was used to visualize data on a heat map and allow one dimensional clustering across features (characters).

For all other data, analysis of variance (ANOVA) was performed using SPSS software (SPSS Inc., Chicago, IL, USA). Significant differences were calculated by Tukey’s mean test. Differences at P ≤ 0.05 were considered significant.

## Supplementary information

Supplementary Tables and Figures

Supplementary Table 1

Supplementary Table 4

## Data Availability

All key data are provided in the Supplementary Files. Further primary datasets generated during and/or analysed during the current study are available from the corresponding author on reasonable request.
