# Supplementary material for: A complex interaction between pre-harvest and post-harvest factors determines fresh-cut melon quality and aroma
Source: Sci Rep. 2019 Feb 26;9:2745. doi: 10.1038/s41598-019-39196-0 (PMC6391468; doi:10.1038/s41598-019-39196-0)
Supplement: Supplementary file 1 — Supplementary Tables and Figures [file 41598_2019_39196_MOESM1_ESM.pdf]

A complex interaction between pre-harvest and post-harvest factors determines fresh-cut melon quality and aroma.

Natasha D. Spadafora[1-2#], Giacomo Cocetta[3#], Marina Cavaiuolo[3^], Roberta Bulgari[3] Rakhee Dhorajiwala[1], Antonio Ferrante[3], Anna Spinardi[3], Hilary J. Rogers[1*], Carsten T. Müller[1].

SUPPLEMENTARY TABLES S2& S3
SUPPLEMENTARY FIGURES S1- S5

Supplementary Table S2 Weather data for 2013 and 2014 for area near Milan, Italy.

year	Mean max	Mean min
2013	32.2	17.7
2014	30.0	16.5
P value	0.01049	0.0318

Supplementary Table S3 : All primers used for PCR in this study

Gene	Primer sequence (5'→3')
CmActin For	GTTCACAACCACTGCCGAAC
CmActin Rev	GAAAAGAACTTCGGGGCAAC
CmAAT1 For	TGTCTGTGGGTTTGAATTGACT
CmAAT1 Rev	ATGAAACAAATCTCATTTCGATGGG
CmAAT2 For	TATGCTTGCCACCTCCTGAC
CmAAT2 Rev	ATGAAACAAATCTCATTTCGATGGG
CmERF71 For	CGCCTACAAAAACCCCCTCT
CmERF71 Rev	GTCACGGCGAGGGATTAAGT
CmERF106 For	GGAGTAGGGTTTGGTTGGGG
CmERF106 Rev	GTCAGACTTTCCGGCATCCA
CmTINY For	AAGAACAGAGCCCTTCTCCG
CmTINY Rev	AGTCCCTAACCAAATGCGGG

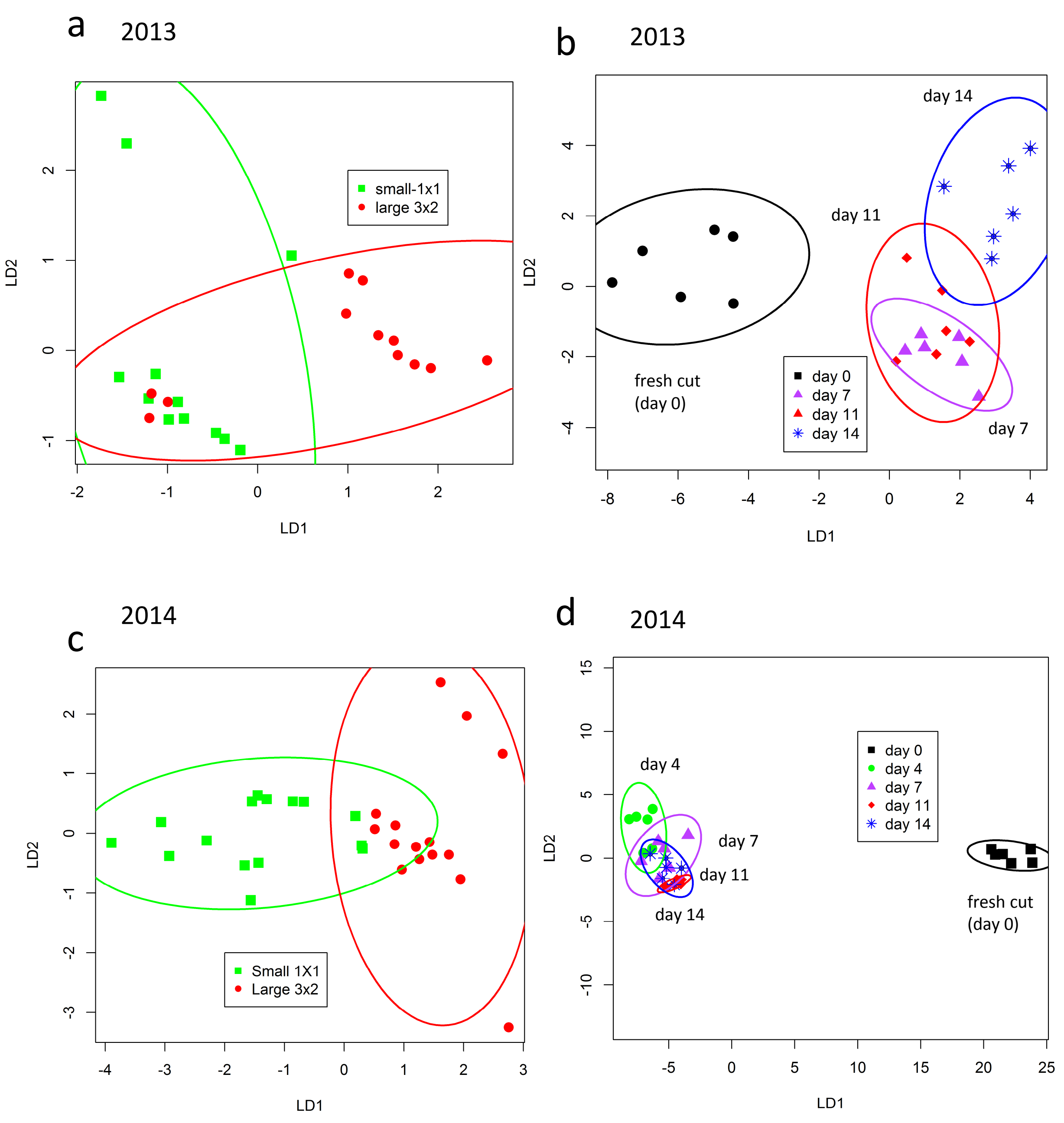

Supplementary Fig. S1. Canonical Analysis of Principal coordinates related to cut size and time of storage separately based on all VOCs from 2013 and 2014 melon using TD-GC-TOF-MS: (a) 2013 cut size, 2 x 3 (L) or 1 x 1 (S) cm, (b) 2013 time of storage at 4 °C, (c) 2014 cut size, 2 x 3 (L) or 1 x 1 (S) cm, (d) 2014 time of storage at 4 °C Each ellipse represents the 95% confidence interval. The plots use LD1 and LD2 (a) and (c) with a percentage of correct classification = 83.3% and 86.7% respectively (n=12)); (b) and (d) with a percentage of correct classification of 83.3% and 70 respectively (n=6).

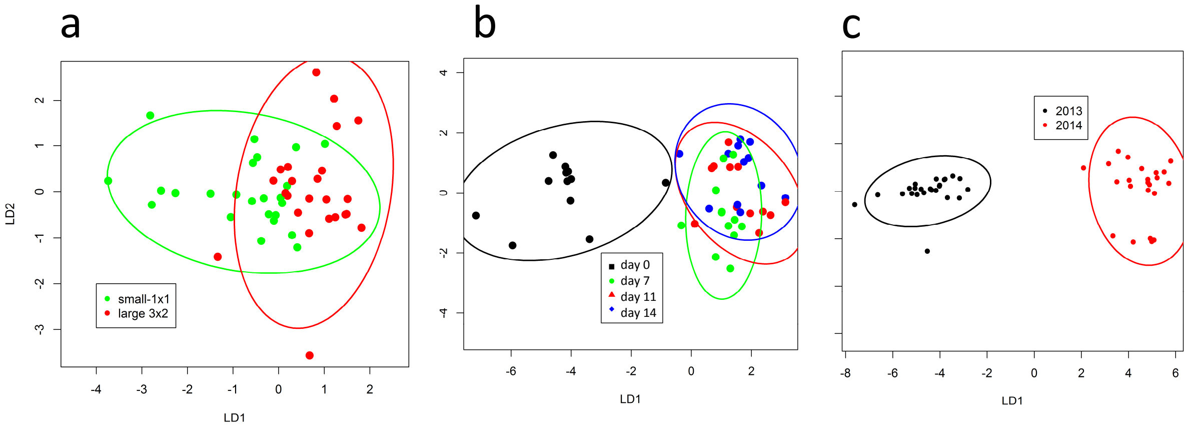

Supplementary Fig. S2. Canonical Analysis of Principal coordinates related to cut size and time of storage separately based on all shared VOCs from 2013 and 2014 melon using TD-GC-TOF-MS: (a) cut size, 2 x 3 (L) or 1 x 1 (S) cm, (b) time of storage at 4 °C, (c) season (2013 or 2014). Each ellipse represents the 95% confidence interval. The plots use LD1 and LD2 with a percentage of correct classification of (a) 62.5%, (b) 62.5% and (c) 100%

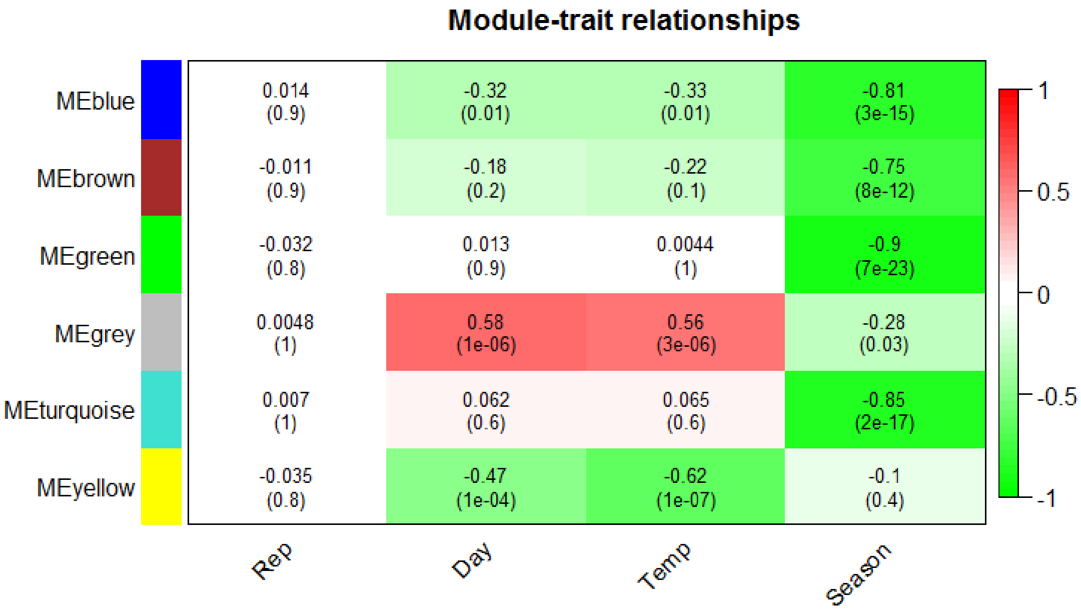

Supplementary Figure S3 . WCNA modules (using softpower 12 and module size 3) from an analysis of the shared VOCs between the two seasons across two temperatures (4 ºC and 20 ºC) and across 2 weeks of storage; the score and significance (*P* values in brackets) are according to a Pearson analysis.

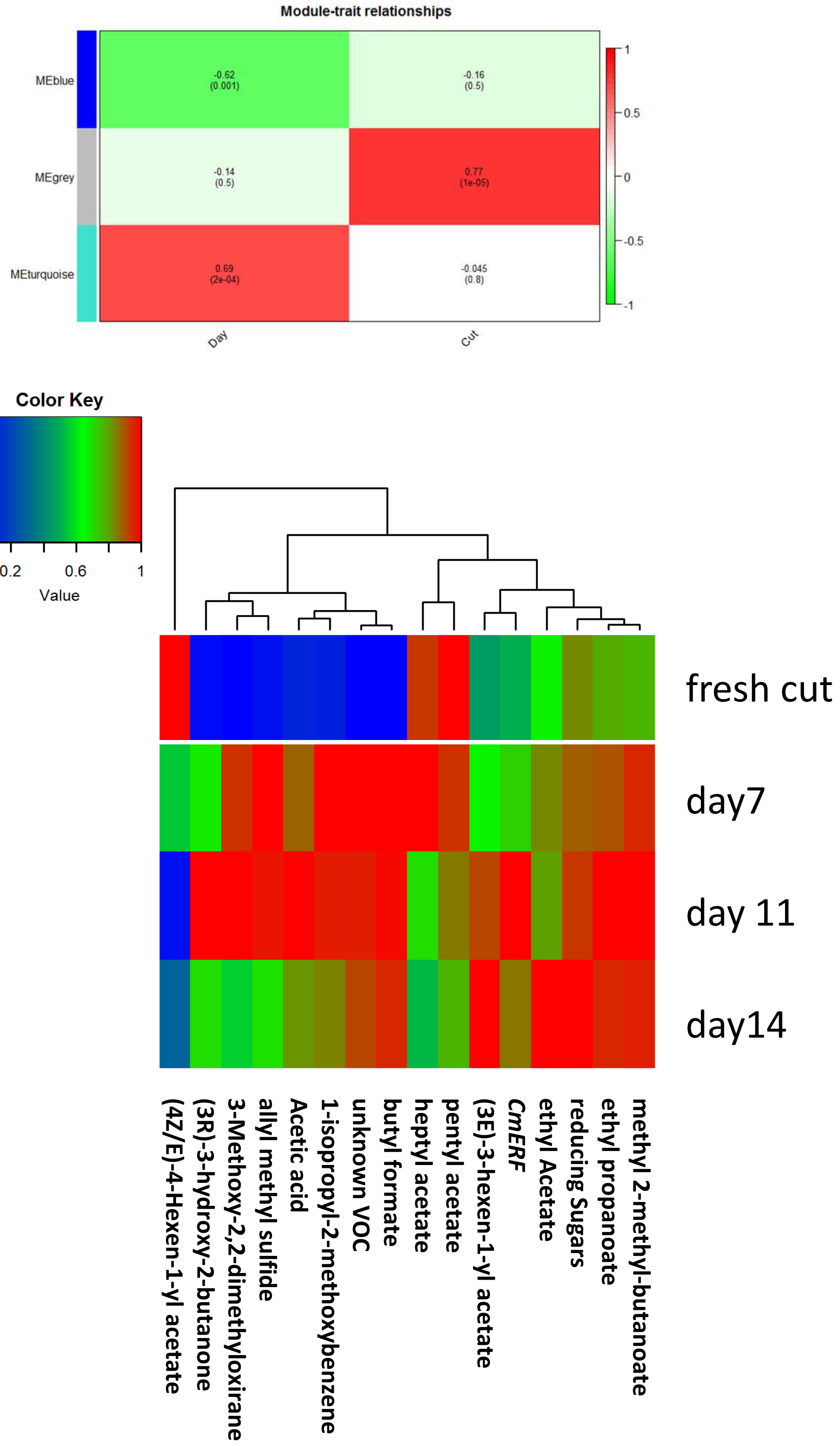

Supplementary Figure S4 . Multi-trait correlation analysis of the shared VOCs between the two seasons across two cut sizes, with metabolites and gene expression data from the 2014 season and across 2 weeks of storage: (a) WCNA modules (using softpower 7 and module size 6) from a multi-trait analysis; the score and significance (*P* values in brackets) are according to a Pearson analysis. **(b)** Multi-trait analysis heat map and hierarchical clustering based on day of storage using characters grouped into the Blue and Turquoise modules in (a) and which were significantly correlated with days of storage. Blue indicates low, green intermediate and red high abundance of the character (transcript, metabolite or VOC).

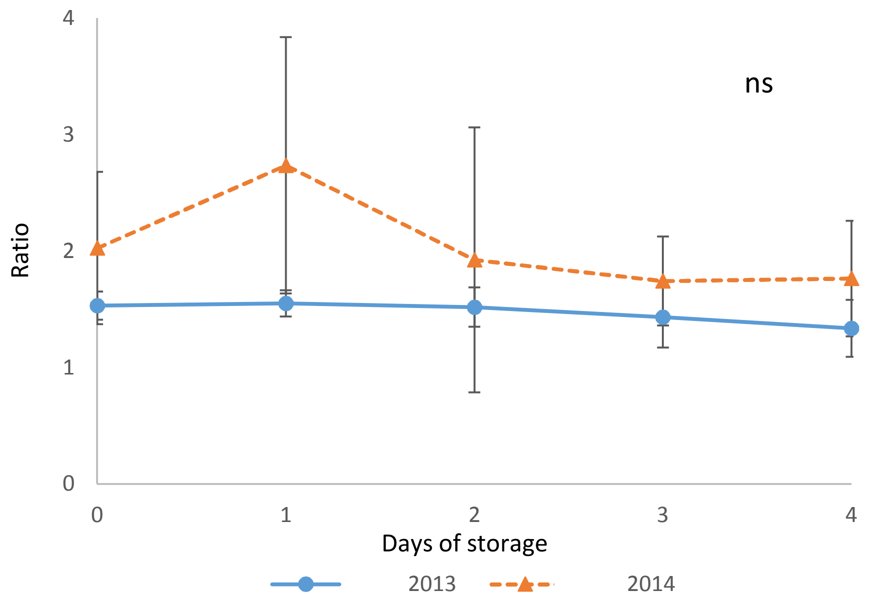

Supplementary Figure S5. Change in ratio of acetate esters to non acetate esters during storage of fresh cut melon pieces at 20 ^{o}C; mean $\pm$ SD; n=3; ns indicates no significant differences $P < 0.05$.

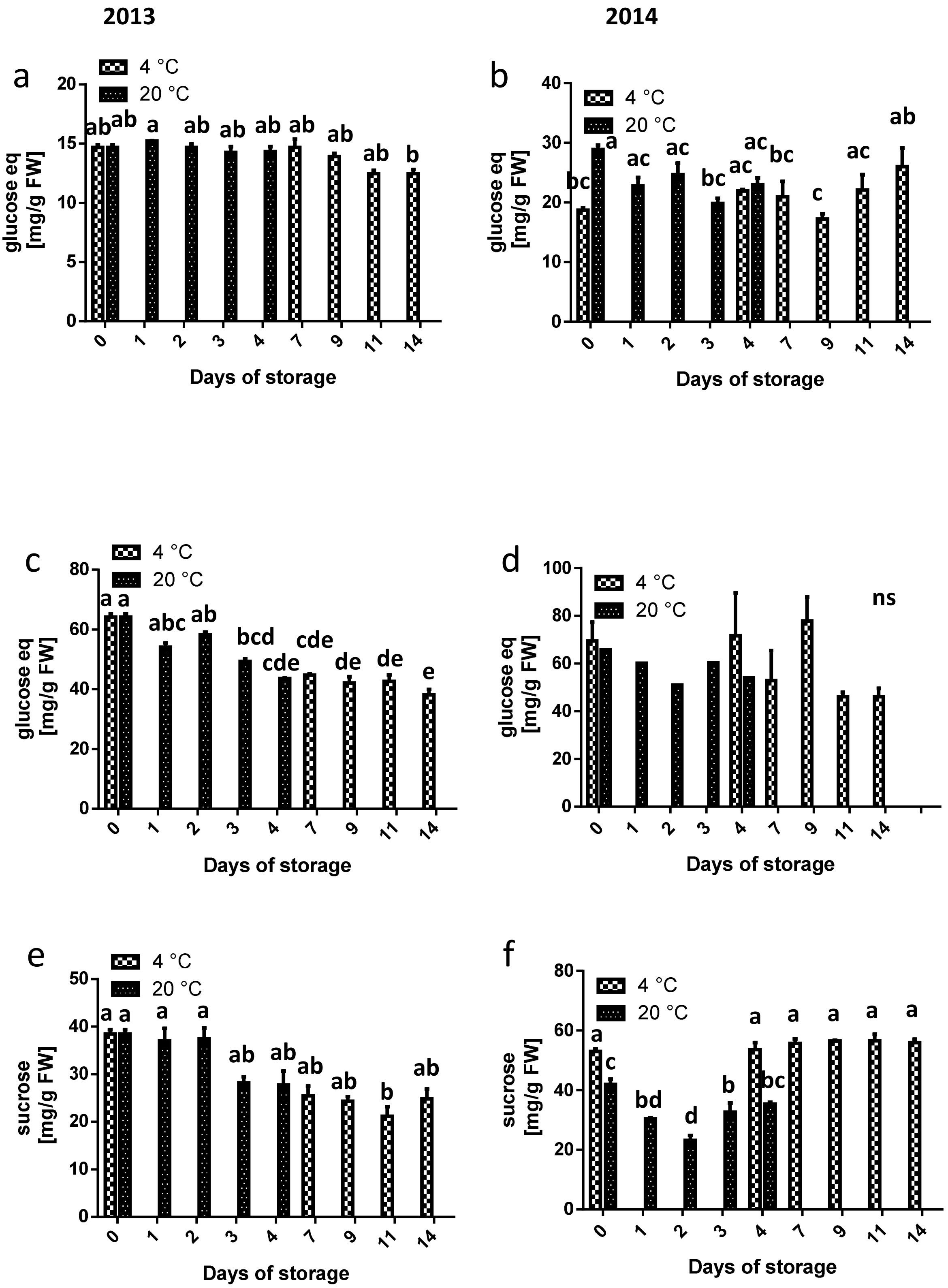

Supplementary Figure S6. Changes in sugars over storage time with temperature of storage and across the two seasons. Reducing sugars (a) 2013 (b) 2014; total sugars (c) 2013 (d) 2014; sucrose (e) 2013 (f) 2014; n=3 bars indicate ± SE. Different letters indicate statistically significant differences (P < 0.05)
